# Tailoring the Interface with a Multifunctional Ligand for Highly Efficient and Stable FAPbI_3_ Perovskite Solar Cells and Modules

**DOI:** 10.1002/advs.202301603

**Published:** 2023-05-11

**Authors:** Fuqiang Li, Xiaofeng Huang, Chaoqun Ma, Junpeng Xue, Ying Li, Danbi Kim, Hyun‐Seock Yang, Yuanyuan Zhang, Bo Ram Lee, Junghwan Kim, Binghui Wu, Sung Heum Park

**Affiliations:** ^1^ Department of Physics Pukyong National University Busan 48513 South Korea; ^2^ Institute of Energy Transport and Fusion Research Pukyong National University Busan 48513 Republic of Korea; ^3^ College of Chemistry and Chemical Engineering Pen‐Tung Sah Institute of Micro‐Nano Science and Technology Xiamen University Xiamen 361005 China; ^4^ School of Materials Science and Engineering University of Science and Technology Beijing Beijing 100083 China; ^5^ School of Science Jiangsu Key Laboratory of Function Control Technology for Advanced Materials Jiangsu Ocean University Lianyungang Jiangsu 222005 China

**Keywords:** interfacial engineering, multiple bonds effect, perovskite solar cells, solar modules

## Abstract

Perovskite solar cells (PeSCs) using FAPbI_3_ perovskite films often exhibit unfavorable phase transitions and defect‐induced nonradiative interfacial recombination, resulting in considerable energy loss and impairing the performance of PeSCs in terms of efficiency, stability, and hysteresis. In this work, a facile interface engineering strategy to control the surface structure and energy‐level alignment of perovskite films by tailoring the interface between the FAPbI_3_ film and hole‐transporting layer using 4‐hydroxypicolinic acid (4HPA) is reported. According to density functional theory studies, 4HPA has prominent electron delocalization distribution properties that enable it to anchor to the perovskite film surface and facilitate charge transfer at the interface. By enabling multiple bonding interactions with the perovskite layer, including hydrogen bonds, Pb—O, and Pb—N dative bonds, 4HPA passivation significantly reduces the trap density and efficiently suppresses nonradiative recombination. The obtained perovskite films exhibit superior optoelectronic properties with improved crystallinity, pure *α*‐phase FAPbI_3_, and favorable energy band bending. Following this strategy, 4HPA post‐treatment PeSCs achieve a champion power conversion efficiency of 23.28% in 0.12 cm^2^ cells and 19.26% in 36 cm^2^ modules with excellent environmental and thermal stabilities.

## Graphical abstract

The surface post‐treatment of FAPbI_3_ perovskite film with multifunctional molecule 4‐hydroxypicolinic acid (4HPA) shows excellent optoelectronic properties with improved crystallinity, pure *α*‐phase FAPbI_3_, and favorable energy band bending. The 4HPA post‐treated PeSC achieves a champion power conversion efficiency of 23.28% in 0.12 cm^2^ cells and 19.26% in 36 cm^2^ modules, with excellent environmental and thermal stabilities.

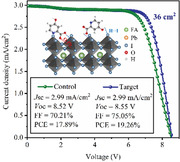

## 1 Introduction

Metal halide perovskite solar cells (PeSCs) have gained considerable attention during the past decade because of their superior advantages such as excellent photovoltaic properties,^[^ ^1^ ^]^ low‐cost solution processability,^[^ ^2^ ^]^ and unprecedented rise in their power conversion efficiency (PCE).^[^ ^3^ ^]^ Indeed, the latest certified PCE for single‐junction PeSCs has now reached 25.7%,^[^ ^4^ ^]^ exceeding that of top‐level polycrystalline silicon solar cells, making them highly attractive for commercialization. Despite the tremendous advancements made toward improving efficiency, the long‐term stability and restricted scalable fabrication protocols remain major hurdles in their industrialization.^[^ ^5^ ^]^ Specifically, most PeSC materials use organic formamidinium (FA) or methylammonium (MA) cations in combination with inorganic PbI_3_ perovskite components.^[^ ^6^ ^]^ Among them, FAPbI_3_ is generally preferred over MAPbI_3_ because of its higher thermal stability,^[^ ^7^ ^]^ superior charge carrier transport capabilities,^[^ ^8^ ^]^ and smaller bandgap energy that extends into the near‐infrared range.^[^ ^9^ ^]^ Nevertheless, at room temperature, the phase transformation of the photovoltaically active black *α*‐phase of FAPbI_3_ to the undesirable but more stable yellow *δ*‐phase, a non‐perovskite material, severely limits practical PeSC applications based on FAPbI_3_.^[^ ^10^ ^]^ In addition, the solution‐processed FAPbI_3_ film inevitably yields a significant number of defects, especially at the surfaces and grain boundaries (GBs), owing to the loss of organic components during thermal annealing and the heterogeneity of polycrystals.^[^ ^11^ ^]^ These defects act as trap sites in the charge carriers transport and collection process, thereby degrading the efficiency and long‐term operational stability of PeSCs.^[^ ^12^ ^]^ Moreover, these defects hinder the scalability of PeSCs to modules, ultimately limiting their potential for commercialization.^[^ ^5b^ ^]^

Researchers have investigated compositional optimization and additive incorporation of FAPbI_3_ materials to address these issues.^[^ ^13^ ^]^ However, although these diverse approaches have achieved tremendous success, improvements in stability and photoelectric efficiency are often accompanied by unintended consequences.^[^ ^14^ ^]^ For instance, the alloying reaction of MA and Br in FAPbI_3_ gives rise to various issues, including low thermal stability due to MA molecules,^[^ ^15^ ^]^ phase separation due to the presence of mixed halides,^[^ ^16^ ^]^ and reduced photon absorption, resulting in a low current density due to undesirable widening of the bandgap.^[^ ^17^ ^]^ Optimization of the growth of FAPbI_3_ crystals by introducing additives, including Pb(SCN)_2_,^[^ ^18^ ^]^ 1‐cyclohexyl‐2‐pyrrolidone, and thiosemicarbazide,^[^ ^19^ ^]^ is incompatible with low‐cost scalable coating processes such as blade‐coating, which remains a challenge for the scale‐up manufacture of large‐area perovskite films.^[^ ^20^ ^]^ These negative consequences significantly restrict the large‐scale development of FAPbI_3_‐based perovskite device applications.

Meanwhile, interface modification has proven to be an effective and straightforward strategy for reducing defect density and improving charge transport without the need for complex fabrication processes.^[^ ^21^ ^]^ Up to now, achieving efficient and stable PeSCs often involves using surficial post‐treatment with alkylammonium halides, and numerous compounds have been assessed.^[^ ^22^ ^]^ After treatment with alkylammonium halides, a secondary 2D perovskite layer is typically formed on top of the primary perovskite layer, which improves device stability.^[^ ^23^ ^]^ Unfortunately, surface 2D perovskite layers often exhibit a tenacious in‐plane orientation and high exciton binding energy, which may limit the impact of passivation and impede charge transfer, especially in the presence of larger spacer cations.^[^ ^24^ ^]^ This prompted You et al. to eliminate the annealing process after post‐treatment to replace the 2D perovskite layer with a thin phenylethylammonium iodide (PEAI) layer, resulting in more efficient surface passivation.^[^ ^25^ ^]^ Consequently, they achieved a certified PCE of more than 23% using a combination of *π*‐conjugated phenyl rings promoting charge transfer, amine coordination reducing Pb^2+^ interstitials, and iodide ions filling iodine vacancies. However, the PCEs of these devices still decreased at higher working temperatures because of the conversion of PEAI to 2D PEA_2_PbI_4_. In addition, long alkyl chains in the modifier can create an unnecessary charge barrier between the hole transfer layer (HTL) and perovskite layer.^[^ ^26^ ^]^ Therefore, there is a great need for a short chain‐based agent or related new hybrid structure that can withstand higher temperatures without forming 2D perovskite and can effectively passivate the surface defects of perovskite to achieve high‐performance PeSCs and modules.

In this study, we selected a pyridine‐based multifunctional ligand, 4‐hydroxypicolinic acid (4HPA), to tailor the interface between the FAPbI_3_ perovskite and the HTL. There are multiple advantages to using 4HPA to improve the photovoltaic performance of perovskite devices. First, 4HPA exhibits significant electron delocalization properties through its unique conjugated *π* system, which enhances the effectiveness of charge transfer through the ligand compared to ligands containing alkyl chains. Second, the *π*‐conjugated aromatic backbone in 4HPA has strong steric hindrance, which allows 4HPA to be anchored to the perovskite surface in a certain orientation, which is beneficial for improving the charge transfer at the interface. Third, the ligand has good rigidity and high thermal stability, contains a carbonyl group (C=O) and hydrogen bonding donors (—OH), which can passivate ionic defects through coordination and hydrogen bonding. To further investigate the correlation between ligand molecular structure and device performance, we also compared the effects of 2‐picolinic acid (PA) and its isomer 5‐hydroxypyridine carboxylic acid (5HPA) on device performance (Figure S14, Supporting Information). As a result, the as‐fabricated devices modified with optimized 4HPA showed a remarkable improvement in performance, with a PCE of 23.28%, an open‐circuit voltage (*V*_oc_) of 1.15 V, and a fill factor (FF) of 80.11%, outperforming the control device (19.23%). In addition, the devices demonstrated enhanced long‐term stability under ambient conditions and high temperature. Furthermore, a FAPbI_3_‐based perovskite module with a total area of 36 cm^2^ exhibited a high efficiency of 19.26%.

## 2 Results and Discussion

**Figure 1:**
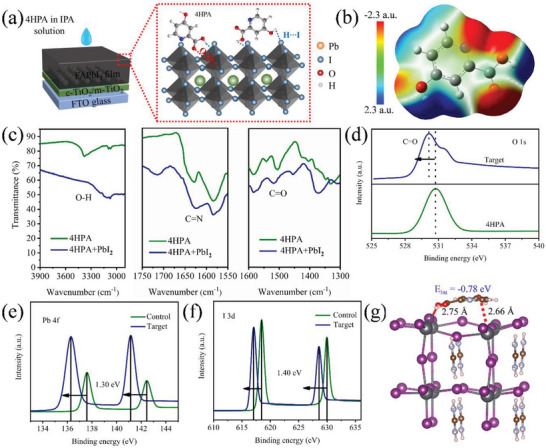
a) Schematic diagram of the interaction between 4HPA and the FAPbI_3_ perovskite film, including the coordination of the carbonyl unit with Pb, and the hydrogen bond between the hydroxyl unit and I. b) Visualization of the ESP result of 4HPA; c) FTIR spectra in the fingerprint regions of 4HPA powder and 4HPA: PbI_2_ blend. XPS profiles of d) O 1s for pure 4HPA and 4HPA‐treated perovskite film and e) Pb 4f and f) I 3d peaks of control and target films. g) Optimized surface structure of interaction between FAPbI_3_ and 4HPA molecule. The calculated binding energy between the FAPbI_3_ and 4HPA molecule is indicated in the figure.

To test the potential of the 4HPA ligand and assess its role in enhancing the photovoltaic performance of perovskites, a solution of 4HPA in isopropyl alcohol was spin‐coated on top of the annealed FAPbI_3_ perovskite film (**Figure** **1**a). In addition, a pristine FAPbI_3_ film was fabricated using a one‐step method for reference purposes (for experimental details, see Supporting Information). Hereinafter, the pristine composition (FAPbI_3_) and the perovskite coated with 4HPA (FAPbI_3_/4HPA) are referred to as the control and the target material, respectively. Based on the statistical analysis of PeSC efficiencies treated with various concentrations of 4HPA, we determined that the optimal concentration is 1.0 mg mL^−1^. We first used density functional theory (DFT) to perform quantum chemical calculations and visualize the frontier orbitals of 4HPA. As illustrated in Figure S1 (Supporting Information), the HOMO and LUMO of 4HPA were found to be delocalized throughout the entire molecule because of the balanced electron‐withdrawing nature of the Lewis base carbonyl (C=O) unit. The simulated electrostatic potential (ESP) surfaces of 4HPA provided a 3D view of the charge distribution of the molecule, as shown in Figure 1b. A negative ESP was observed on the pyridine ring. Conversely, a stronger negative ESP was observed on the carbonyl and two hydroxyl end groups, which enhanced its passivation ability. Therefore, pyridine and carbonyl groups are expected to passivate the uncoordinated Pb^2+^ in FAPbI_3_ through coordination between the Lewis acid and base.

To confirm the formation of Lewis adducts of Pb and O or N, we carried out UV–vis absorption measurements for neat 4HPA and mixed 4HPA:PbI_2_ solutions. Typically, the formation of coordination bonds between metal ions and organic ligands affects the conjugated structure of chromophores, resulting in shifts in the absorption peaks.^[^ ^27^ ^]^ When dissolved in an *N*, *N*‐dimethyl formamide (DMF) solution, 4HPA displays a broad absorption band in the 250–400 nm range, with two characteristic peaks at 282 and 360 nm, as shown in Figure S3 (Supporting Information). Upon mixing 4HPA with PbI_2_ in the same DMF solution, the resulting mixture exhibits significantly stronger and blueshifted absorption (two peaks at 471 and 353 nm) than the neat 4HPA solution. The observed changes can be attributed to the Lewis base–acid interaction and hydrogen bonding between PbI_2_ and 4HPA (N/C=O with Pb^2+^, —OH with I^−^), as well as a weakening of the push‐pull effect of 4HPA. Fourier transform infrared (FTIR) spectra were recorded to further confirm the interaction mechanism to analyze the 4HPA powder and the 4HPA:PbI_2_ mixture (Figure 1c). The neat 4HPA powder exhibited symmetric stretching vibrations of the C=O and —OH bonds at 1506 and 3380 cm^−1^, respectively. The mixture of 4HPA with PbI_2_ broadened and weakened the bands representing the vibration of the —OH bond (shifted by 271 cm^−1^). Conversely, the vibrational signal of the C=O groups shifted by 13 cm^−1^ to higher wavenumbers, suggesting the presence of significant interactions (dative covalent bonds and hydrogen bonds) between the solid 4HPA and PbI_2_. The shift of the C=O stretching vibration peak to higher wavenumbers was also observed in the target film (Figure S5, Supporting Information). The substantially altered —OH vibration signal suggests that the —OH group plays a prominent role in Lewis base–acid interactions. In addition, the shift in the vibrational bands of C=O groups in the 4HPA:PbI_2_ mixture implies that the push‐pull structure of 4HPA (—OH → C=O) facilitates the coordination of C=O and Pb^2+^. According to previous reports,^[^ ^28^ ^]^ we estimated that the —OH groups formed hydrogen bonds with iodide anions, which contributed to the binding of the C=O group to the uncoordinated Pb^2+^ defects in the FAPbI_3_ films. In comparison, the vibrational signal of the C=N group insignificantly shifted after 4HPA was mixed with PbI_2_, indicating weaker interactions between C=N and PbI_2_.

X‐ray photoelectron spectroscopy (XPS) measurements further confirmed the electronic interaction between 4HPA and perovskite. The O 1s peak of the 4HPA molecule is characterized by a binding energy of 532.4 eV (Figure 1d). However, upon coating 4HPA onto the perovskite film, the position of the peak shifted to 530.16 eV, indicating that C=O as a Lewis base strongly binds to the uncoordinated Pb^2+^ ions to form Lewis adducts, and the increased activation energy of ions can reduce ion migration.^[^ ^29^ ^]^ Computational confirmation of a stable adsorbate structure was achieved through density functional theory (DFT) calculations, which identified a surface binding energy of −0.78 eV (Figure 1g). In this structure, the oxygen from the carbonyl group of 4HPA binds to the uncoordinated Pb^2+^ on the surface, while the hydroxyl groups form hydrogen bonds with the adjacent surface iodides (Figure S8, Supporting Information). The —OH group forms intermolecular hydrogen bonds with I^−^, which slows down the crystallization rate, immobilizes the I^−^ ions, and inhibits the diffusion of metal ions, resulting in further improvement of device stability.^[^ ^30^ ^]^ Moreover, the peak of N 1s appears to have a significant shift (more than 0.40 eV) toward a lower binding energy, as shown in Figure S7 (Supporting Information).^[^ ^31^ ^]^ The 4HPA molecule contains an electron‐rich N atom with a coordinated lone pair of electrons. As a result of the electrostatic attraction, the lone pair electrons establish dative covalent bonds with the positive charge on the metal ion and cause the positive charge to become delocalized within the pyridine molecule. A shift in the binding energy is expected due to charge transfer between the perovskite and the Lewis base. As shown in Figure 1e,f, the binding energies of Pb (Pb 4f_7/2_, Pb 4f_5/2_) and I (I 3d_5/2_, I 3d_3/2_) in the target film are moved to lower positions than that of the control film (1.30 and 1.40 eV for Pb 4f and I 3d, respectively). This shift in binding energies suggests that the interaction between Pb and I is weakened after treatment with 4HPA, likely due to the bonding of 4HPA with Pb and I. Simultaneously, the treatment effectively passivated uncoordinated lead and halogen defects, resulting in an improvement in the crystal quality of the perovskite films.^[^ ^32^ ^]^

**Figure 2:**
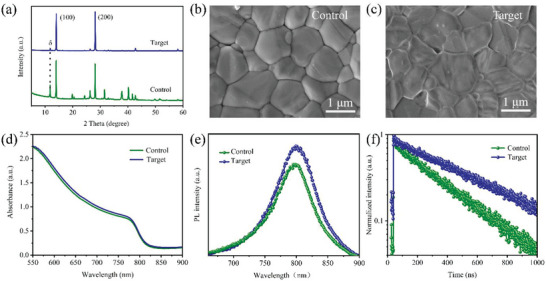
a) XRD patterns of the control and target films. Top‐view SEM images of the b) control and c) target perovskite films. d) UV–vis absorption, e) PL, and f) TRPL spectra of the corresponding perovskite films deposited on the glass substrates.

X‐ray diffraction (XRD) measurements were performed to study the effect of 4HPA post‐treatment on the crystallization properties of FAPbI_3_ films. The two dominant peaks at ≈14° and 28° in the XRD pattern were assigned to the characteristic (100) and (200) crystal planes of the *α*‐phase FAPbI_3_.^[^ ^10b^ ^]^ In the control perovskite film, the peaks corresponding to the *α*‐phase perovskite were dominant, but the *δ*‐phase FAPbI_3_ peak at 11.4° was present (**Figure** **2**a). In the target film, the characteristic peak corresponding to *δ*‐phase FAPbI_3_ (non‐perovskite phase) decreased significantly after treatment with 4HPA. Furthermore, the diffraction intensities of the (100) and (200) peaks are enhanced. These findings demonstrate that the interaction between 4HPA and PbI_2_ promotes the reconstruction of the perovskite layer and effectively suppresses the formation of *δ*‐phase FAPbI_3_. Moreover, the absence of peaks in the low‐angle area implies that the post‐treatment is unrelated to the 2D perovskite formation. To explore the impact of 4HPA on the morphology of the FAPbI_3_ films, top‐view scanning electron microscopy (SEM) measurements were conducted. The control film without the 4HPA treatment exhibited obvious GBs on the surface of the FAPbI_3_ film (Figure 2b). A uniform distribution of all relevant elements, including O, present only in 4HPA suggests that 4HPA evenly covers the perovskite thin film (Figure S9, Supporting Information). Upon treatment with 4HPA, the film displays a compact morphology with obvious stacking layers of 4HPA on the surface of the FAPbI_3_ film (Figure 2c). Simultaneously, the resultant GBs of the surface perovskite became less pronounced, benefitting from the anchoring of 4HPA on the surface. After 4HPA modification, unchanged perovskite grain size indicates that no recrystallization process occurred (Figure S10, Supporting Information). As illustrated in Figure S11 (Supporting Information), the root surface roughness of the perovskite films was compared using atomic force microscopy. The roughness of the target perovskite film decreased, with a root mean square (RMS) of 59.1 nm compared to that of the control film of 76.1 nm. The smooth surface of the perovskite film helps establish a uniform HTL on the surface, thereby facilitating carrier extraction and suppressing charge recombination at the interface.

To track the enhancement of charge extraction and reduction of charge recombination, the optical properties of the perovskite films were explored. Following the 4HPA treatment, the absorption of the perovskite thin film increased (Figure 2d), which was attributed to the enhanced morphology and crystallization of the film. Steady‐state photoluminescence (PL) spectra of the perovskite thin films deposited on glass substrates were recorded. Figure 2e shows that the PL intensity of the target film is noticeably higher than that of the control FAPbI_3_ film, indicating that nonradiative recombination was suppressed because of the effectively reduced surface charge traps.^[^ ^33^ ^]^ In addition, time‐resolved photoluminescence (TRPL) measurements were performed to elucidate the contribution of the enhanced PL lifetime. As shown in Figure 2f and Table S2 (Supporting Information), the TRPL lifetimes of the target thin film are extended. This indicates that treatment with 4HPA can effectively passivate defects in the perovskite film, thereby promoting charge carrier transport with reduced nonradiative recombination losses, resulting in superior performance in both *V*oc and FF of the devices.

**Figure 3:**
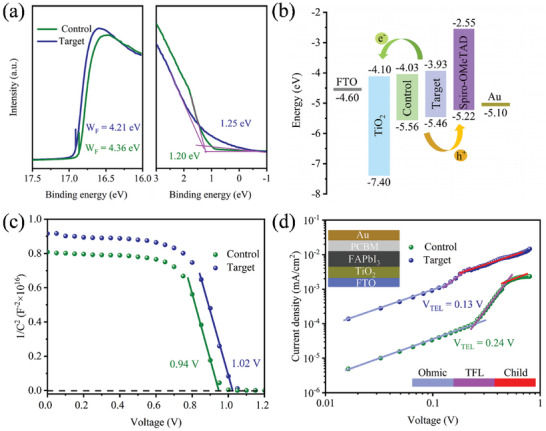
a) UPS results of the corresponding perovskite thin films. b) Band alignment of the PeSCs after 4HPA post‐treatment. c) Mott–Schottky measurements for the corresponding PeSCs. d) Dark *J*–*V* characteristics of the control and target devices. The inset shows the device structure.

The influence of 4HPA on the electronic properties of perovskites was elucidated using UV photoelectron spectroscopy (UPS). The corresponding UPS spectra of the secondary electron cut‐off region and valence band edge region are shown in **Figure** **3**a. According to these UPS results, the work functions of the control and target films are calculated to be 4.36 and 4.21 eV, respectively, and their valence band maxima were determined to be 5.56 and 5.46 eV, respectively. Figure 3b depicts the energy‐level diagrams for the films based on the UPS and Tauc plots (Figure S13, Supporting Information), revealing that the valence band and conduction band edges of the perovskite migrated higher after the 4HPA post‐treatment. This optimized energy‐level alignment allows the photogenerated holes to be extracted from the perovskite layer to the HTL with slight energy loss and allows electrons to be bounced back at the interface.^[^ ^34^ ^]^ Therefore, PeSCs fabricated using 4HPA‐treated films are expected to have a higher *V*_oc_.^[^ ^35^ ^]^ To evaluate this idea, the *C* ^−2^ versus *V* characteristics of the corresponding devices were obtained by capacitance–voltage measurements (Figure 3c). The built‐in voltages (*V*_bi_) were estimated as 0.94 and 1.02 V for the control and target devices, respectively. The highest *V*_bi_ of the 4HPA treated device is usually related to the highest *V*_oc_. Then, the dark space‐charge‐limited currents were measured for the control and 4HPA post‐treated devices. The defect density was calculated on the grounds of the equation of *N*_d_ = (2*εε*_0_ *V*_TFL_)/(*eL* ^2^), where *e* is the elementary charge, *L* is the thickness of the perovskite thin film, *ε*_0_ is the permittivity in vacuum (8.85 × 10^12^ F m^−1^), and *ε* is the relative dielectric constant of FAPbI_3_ (46.9). The determined *V*_TFL_ value of the target device decreased from 0.24 V for the control device to 0.13 V (Figure 3d). Based on these values, the electron defect density *N*_d_ of the target device was calculated to be 2.70 × 10^15^ cm^−3^ and that of the control was 4.98 × 10^15^ cm^−3^. The reduced defect state density in the 4HPA‐treated films compared to that in the control films is precisely due to the anchoring of uncoordinated ions (Pb^2+^ and I^−^) and the subsequent retardation of ion migration by 4HPA passivation. A lower defect density is advantageous for a steady device output and suppression of hysteresis. Furthermore, because the defects of the perovskite films are mostly concentrated at the GBs, the ligand can effectively cover the surface and penetrate the surface at the GBs during treatment, thereby improving the *V*_oc_ of the devices.

**Figure 4:**
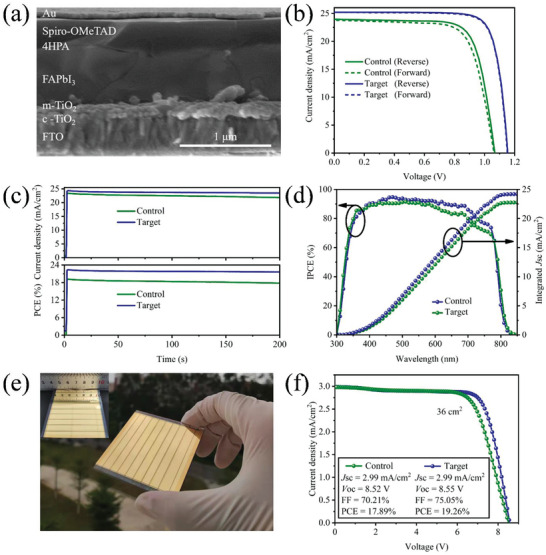
a) Cross‐sectional SEM image of the target PeSC. b) *J*–*V* curves of the control and target champion devices for different scan directions. c) Steady‐state power output of the corresponding devices biased at the MPP. d) IPCE spectra and integrated photocurrent density of the corresponding devices. e) Photographic image of a laser‐etched 6.0 × 6.0 cm perovskite solar module. f) *J*–*V* curves of the perovskite modules.

To further assess the effect of 4HPA on the photovoltaic characteristics of PeSCs, devices with FTO/TiO_2_/perovskite/4HPA/Spiro‐OMeTAD/Au planar configuration were fabricated, as depicted in **Figure** **4**a. Figure S15 (Supporting Information) presents a statistical distribution diagram of the performance of the devices treated with various concentrations of 4HPA, while Table S3 (Supporting Information) lists the corresponding photovoltaic parameters. The optimal performance of the target device was determined at a concentration of 1.0 mg mL^−1^. The control device demonstrated an average short‐circuit current density (*J*_sc_) of 23.82 ± 0.43 mA cm^−2^, average *V*_oc_ of 1.05 ± 0.04 V, average FF of 74.84 ± 1.83%, and average PCE of 18.72 ± 0.77%. Conversely, the device treated with 4HPA (1.0 mg mL^−1^) yielded an average *J*_sc_ of 25.08 ± 0.22 mA cm^−2^, an average *V*_oc_ of 1.15 ± 0.02 V, an average FF of 79.18 ± 1.42%, and an average PCE of 22.85 ± 0.43%, respectively. Evidently, both *V*_oc_ and FF are greatly enhanced, along with a slight improvement in *J*_sc_, which is attributed to the efficient passivation of the defects at the surfaces and GBs of perovskite films by 4HPA molecules. Figure 4b depicts the best‐performing *J*–*V* curves for both the control and 4HPA‐treated devices, and **Table** **1** summarizes the relevant photovoltaic characteristics. The control device produced a champion PCE of 19.23% (*J*_sc_ of 24.05 mA cm^−2^, *V*_oc_ of 1.06 V, and FF of 75.21%) in the reverse scan and PCE of 18.26% (*J*_sc_ of 23.79 mA cm^−2^, *V*_oc_ of 1.06 V, and FF of 72.24%) in the forward scan. For comparison, the target device exhibited a significantly enhanced PCE of 23.28% (*J*_sc_ of 25.21 mA cm^−2^, *V*_oc_ of 1.15 V, and FF of 80.11%) in the reverse scan and PCE of 23.15% (*J*_sc_ of 25.18 mA cm^−2^, *V*_oc_ of 1.15 V, and FF of 79.95%) in the forward scan, respectively. Moreover, the hysteresis index (HI) for the control device was 5.04%, whereas that for the target PeSC was 0.56%. A small hysteresis factor implies a more efficient relief of trap states and ion migration in the target device.

**Table 1:** Photovoltaic parameters obtained from the *J*–*V* curves of the control and target champion devices

Device	Scan direction	*J_s_*_c_ [mA cm^−2^]	*V*_oc_ [V]	FF [%]	PCE [%]	HI^a)^ [%]
Control	Reverse	24.05	1.06	75.21	19.23	5.04
	Forward	23.79	1.06	72.24	18.26	
Target	Reverse	25.21	1.15	80.11	23.28	0.56
	Forward	25.18	1.15	79.95	23.15	

^a)^ Hysteresis index (HI) = (PCE_reverse_ − PCE_forward_)/PCE_reverse_.

Figure 4c shows the steady‐state power output at their maximum power point (MPP) for the control and target PeSC devices over 200 s. The control and target PeSC devices responded quickly, and their currents remained at ≈23.4 and 24.3 mA cm^−2^, respectively, confirming the accuracy of the *J*–*V* performance. Particularly noteworthy is that the current density of the control device at the MPP decreased from 23.4 to 21.8 mA cm^−2^ over time, whereas the target device maintained a current density of 23.5 mA cm^−2^ and a stabilized output of 22.6%, demonstrating excellent operational stability. The corresponding incident photon‐to‐electron conversion efficiency (IPCE) spectra demonstrated that the calculated integrated *J*_sc_ value after 4HPA treatment was 24.19 mA cm^−2^, higher than that of the control (22.76 mA cm^−2^), which was consistent with the previous *J*–*V* test results (Figure 4d).

A perovskite solar module with a total area of 36.00 cm^2^ was fabricated to evaluate the scalability of the surface passivation using 4HPA (Figure 4e). Encouragingly, the perovskite solar module based on 4HPA passivation exhibited a *V*_oc_ of 8.55 V, a *J*_sc_ of 2.99 mA cm^−2^, an FF of 75.05%, and a PCE of 19.26% (Figure 4f), whereas the control module shows a PCE of only 17.89% with *V*_oc_ of 8.52 V, *J*_sc_ 2.99 mA cm^−2^, and FF 70.21%. The high module performance results from the uniform perovskite layer, decreased trap density, and suppression of interfacial recombination, proving the viability of the 4HPA passivation technique for scaling up PeSCs.

**Figure 5:**
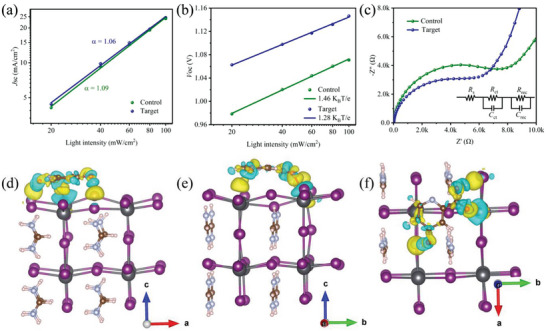
a) *J*_sc_ and b) *V*_oc_ versus the light intensity curves for the control and target PeSCs. c) Nyquist plots of the control and target PeSCs. The inset shows the equivalent circuit diagram. d–f) Differential charge density distribution of the optimized surface structures of the interaction of the perovskite interacting with 4HPA. Yellow and turquoise represent charge accumulation and charge depletion, respectively.

The *J*_sc_ and *V*_oc_ values of the control and target PeSCs were measured under regulated light intensity (*I*) to estimate the extent of trap‐assisted nonradiative recombination and bimolecular radiative recombination. The power law *J*_sc_ ∝ *I^*α*^* describes the relationship between *J*_sc_ and *I*, where *α* is a factor that depends on the degree of bimolecular recombination.^[^ ^36^ ^]^ As shown in **Figure** **5**a, both the control and target devices exhibit similar *α* values that are very close to 1, indicating that bimolecular recombination is negligible owing to the high quality of the perovskites. In addition, according to the following equation, *V*_oc_ should exhibit a linear correlation with the logarithm of light intensity

(1) $$V\mathrm{oc}\;\propto \;n\;\left(\frac{K_{B}T}{e}\right)\log_{10}I+\mathrm{constant}$$

where *n* is the ideality factor, *e* is the elementary charge, *K*_B_ is Boltzmann's constant, and *T* is the thermodynamic temperature. The deviation of the slope from *K*_B_ *T*/e was attributed to trap‐assisted recombination. As shown in Figure 5b, the target device has a smaller slope of 1.28 *K*_B_ *T*/e compared with the 1.46 *K*_B_ *T*/e of the control device, further demonstrating that 4HPA could effectively suppress trap‐assisted recombination. The prominence of both suppressed trap‐assisted recombination and bimolecular recombination revealed that 4HPA converges surface defect passivation and expedites interface charge transfer, thereby lowering energy loss and improving the photovoltaic performance of PeSCs.

Electrochemical impedance spectroscopy (EIS) is an additional helpful technique for gaining insight into the charge transport and transfer processes. Figure 5c shows the Nyquist plots of the EIS measurements performed under dark conditions. Evidently, the target device displayed a lower charge transfer resistance (*R*_ct_) than the control device because of the enhanced interfacial charge transfer assisted by the integrated 4HPA, which should account for the improved FF. In addition, treatment with 4HPA significantly increased the recombination resistance (*R_r_*_ce_) by suppressing charge recombination, as evidenced by the reduced defect density, potentially leading to a higher *V*_oc_. We further applied theoretical calculations to gain deeper insight into the effect of 4HPA on atomic‐scale interface charge transfer. The charge density difference plots are shown in Figure 5d–f. Upon forming the O—Pb coordination bond, the Pb atom gains 0.126 e from 4HPA, and these electrons neutralize the positive charge of unsaturated Pb^2+^, effectively inhibiting charge recombination.

**Figure 6:**
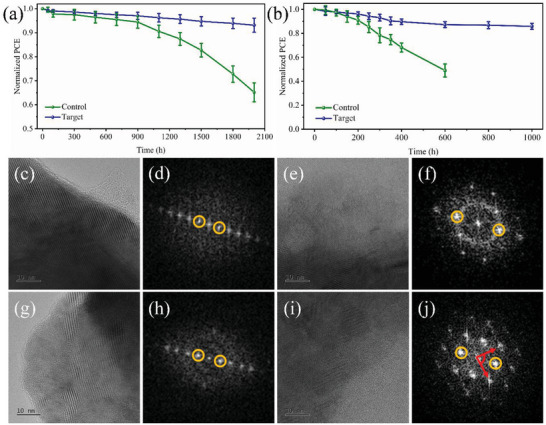
a) PCE decay of the corresponding PeSCs was measured under ambient environmental conditions with 25% humidity in the dark at room temperature. b) PCE decay of the corresponding PeSCs was recorded under heating stress (85 °C) and N_2_ atmosphere. HRTEM of c) fresh target perovskite, e) fresh control perovskite, g) aged (5 min) target perovskite, and i) aged (5 min) control perovskite. Corresponding FFTs of d) fresh target perovskite, f) fresh control perovskite, h) aged (5 min) target perovskite, and j) aged (5 min) control perovskite.

Considering that FAPbI_3_ perovskite undergoes a spontaneous phase transition from *α*‐ to *δ*‐FAPbI_3_ under ambient conditions, we tested the environmental stability of different devices. As a result of the passivation of the decomposition sites in FAPbI_3_ perovskite and the moisture resistance provided by the hydrophobic aromatic rings in 4HPA, the post‐treatment strategy using 4HPA significantly enhances the stability of the *α* phase of FAPbI_3_ perovskite. **Figure** **6**a shows that, with 4HPA passivation, the moisture stability of the unencapsulated FAPbI_3_ devices was considerably improved, maintaining over 90% of the original efficiency for more than 2000 h at an RH of 25 ± 5%, in contrast to less than 70% for the control devices. Post‐treatment with 4HPA increased the contact angle from 70.6° (Figure S16a, Supporting Information) to 82.0° (Figure S16b, Supporting Information) because of the presence of the hydrophobic pyridine ring, demonstrating that 4HPA can enhance the resistance to moisture invasion and improve stability.

We further evaluated the thermal stability of the PeSCs without encapsulation at 85 °C in a nitrogen environment (Figure 6b). The target devices demonstrated exceptional thermal stability, maintaining 85% of their initial PCE after 1000 h. Meanwhile, the control devices retained only 49% of their initial PCE after 600 h, probably because of ion migration inside the devices and the phase instability of bare FAPbI_3_ at high temperatures. GBs are susceptible to thermal stress and can provide channels for the rapid diffusion of atoms and ions. Thus, perovskite was mostly degraded along the GBs. Defect passivation at the GBs by 4HPA suppresses diffusion and enhances thermal stability. A microstructural analysis was performed to further study the role of 4HPA in inhibiting thermal decomposition. High‐resolution transmission electron microscope (HRTEM) was employed to investigate the impact of 4HPA on the phase transformation of perovskites. Specifically, the electron beam (E‐beam) generated by the HRTEM instrument is used as the thermal energy source. The target and control perovskites exhibited FAPbI_3_ layers with different crystallographic orientations, as shown in Figure 6c–f, which also presents the corresponding fast Fourier transforms (FFTs) of the diffraction patterns. Both samples displayed representative spot diffractions (yellow circles) with an interplanar spacing of 6.3 Å, corresponding to the (110) diffraction of FAPbI_3_, as depicted in Figure 6d,f. After exposing the samples to the e‐beam for 5 min, the environmental temperature was increased to ≈130 °C. The HRTEM images and the corresponding FFTs of the diffraction patterns of the aged target and control perovskites are illustrated in Figure 6g–j. Despite the reduced intensity of the (110) diffraction spots in the target perovskite, there was no emergence of new diffraction peaks. In contrast, the control sample exhibited a critical alteration of the FAPbI_3_ layer with new diffraction spots at 3.89 nm^−1^ (2.9 Å) on the FFT. These morphological characteristics indicate that the crystalline perovskite phase was partially transformed into an amorphized phase and that the region had precipitated hexagonal PbI_2_ grains, consistent with previous studies, which found that the thermal degradation of perovskite can generally be considered the reverse process of perovskite film growth.^[^ ^37^ ^]^ Therefore, we speculate that 4HPA acts as a molecular lock when interacting with perovskite, increasing the activation energy required for perovskite decomposition, thus preventing its degradation at high temperatures.

## 3 Conclusion

In summary, we have successfully demonstrated that the pyridine‐based ligand 4HPA can be used as an efficient chemical passivator for the surface of FAPbI_3_ thin films using a post‐treatment approach. Strong interactions between 4HPA and Pb^2+^ ions increased the stabilized phase‐pure FAPbI_3_ perovskite by effectively inhibiting the formation of *δ*‐phase nonperovskite. Moreover, the formation of hydrogen bonds between the 4HPA and I^−^ ions further strengthened the binding and retarded the decomposition of the FAPbI_3_ perovskite films. Multiple interactions of the 4HPA molecule with the perovskite improved the film quality, reduced the trap state density, and suppressed nonradiative recombination. These superior merits enabled a champion PCE of 23.28% in 0.12 cm^2^ cells and 19.26% for 36 cm^2^ modules with excellent environmental and thermal stabilities.

## Conflict of Interest

The authors declare no conflict of interest.

## Supporting information

Supporting Information

## Data Availability

The data that support the findings of this study are available in the Supporting Information of this article.
