# Supplementary material for: Tailoring the Interface with a Multifunctional Ligand for Highly Efficient and Stable FAPbI3 Perovskite Solar Cells and Modules
Source: Adv Sci (Weinh). 2023 May 11;10(21):2301603. doi: 10.1002/advs.202301603 (PMC10375111; doi:10.1002/advs.202301603)
Supplement: Supplementary file 1 — Supporting Information [file ADVS-10-2301603-s001.pdf]

ADVANCED SCIENCE
Open Access

Supporting Information

for *Adv. Sci.*, DOI 10.1002/advs.202301603

Tailoring the Interface with a Multifunctional Ligand for Highly Efficient and Stable $FAPbI_3$ Perovskite Solar Cells and Modules

Fuqiang Li, Xiaofeng Huang, Chaoqun Ma, Junpeng Xue, Ying Li, Danbi Kim, Hyun-Seock Yang, Yuanyuan Zhang, Bo Ram Lee, Junghwan Kim, Binghui Wu and Sung Heum Park**

ADVANCED SCIENCE
Open Access

Supporting Information

for *Adv. Sci.*, DOI 10.1002/advs.202301603

Tailoring the Interface with a Multifunctional Ligand for Highly Efficient and Stable $FAPbI_3$ Perovskite Solar Cells and Modules

Fuqiang Li, Xiaofeng Huang, Chaoqun Ma, Junpeng Xue, Ying Li, Danbi Kim, Hyun-Seock Yang, Yuanyuan Zhang, Bo Ram Lee, Junghwan Kim, Binghui Wu and Sung Heum Park**

Supporting Information

Tailoring the interface with a multifunctional ligand for highly efficient and stable $FAPbI_3$ perovskite solar cells and modules

Fuqiang Li, Xiaofeng Huang, Chaoqun Ma, Junpeng Xue, Ying Li, Danbi Kim, Hyun-Seock Yang, Yuanyuan Zhang, Bo Ram Lee, Junghwan Kim, Binghui Wu and Sung Heum Park**

Fuqiang Li, Junpeng Xue, Ying Li, Danbi Kim, Hyun-Seock Yang, Bo Ram Lee, Sung Heum Park
Department of Physics, Pukyong National University, Busan, 48513, South Korea
E-mail: * spark@pknu.ac.kr

Fuqiang Li, Ying Li, Danbi Kim, Hyun-Seock Yang, Bo Ram Lee, Junghwan Kim, Sung Heum Park
Institute of Energy Transport and Fusion Research, Pukyong National University, Busan 48513, Republic of Korea

Xiaofeng Huang, Binghui Wu
College of Chemistry and Chemical Engineering, Pen-Tung Sah Institute of Micro-Nano Science and Technology, Xiamen University, Xiamen 361005, China
E-mail: * binghuiwu@xmu.edu.cn

Chaoqun Ma
School of Materials Science and Engineering, University of Science and Technology Beijing, Beijing 100083, China

Yuanyuan Zhang
School of Science, Jiangsu Key Laboratory of Function Control Technology for Advanced Materials, Jiangsu Ocean University, Lianyungang, Jiangsu 222005, China

* Corresponding authors.
E-mail addresses: spark@pknu.ac.kr (S.H.Park), binghuiwu@xmu.edu.cn (B.H.Wu)

Experimental section

Materials: Formamidinium iodide (FAI) was purchased from Advanced Election Technology Co., Ltd. 2-methoxyethanol (2ME, anhydrous, 99.8%), titanium diisopropoxide bis(acetylacetonate), isopropyl alcohol (IPA, anhydrous, 99.5%), chlorobenzene (anhydrous, 99.8%), *N*, *N*-dimethylformamide (DMF, anhydrous 99.8%,), and dimethyl sulfoxide (DMSO, > 99.5%) were procured from Sigma-Aldrich. Methylamine hydrochloride (MACl, 98%) was procured from Acros Organics. Lead iodide (PbI_2, 99.999%) was purchased from TCI. Spiro-MeOTAD (99.8%) was purchased from Merck. 4-Hydroxypicolinic acid (CAS: 22468-26-4, chemical structure: C6H5NO3) was obtained from Alfa Aesar. All the purchased chemicals were used as received without further treatment.

Materials Synthesis: Formamidinium lead triiodide ($FAPbI_3$) powder was synthesized by retrograde method. [1] In detail, PbI_2 and FAI (1:1 molar ratio) were dissolved in 2ME (0.8 M) first and then filtered using a polyvinylidene fluoride filter with 0.45 μm pore size. The filtered solution was placed in a flask incubated in an oil bath at 120 °C for 1 h under continuous stirring. The resulting black powder was filtered using a glass filter and dried at 60 °C for 72 hours.

Solar cell fabrication: FTO glass (15 Ω sq^{-1}) substrates were washed by ultrasonication in detergent, deionized water, acetone, and IPA in sequence for 15 min, respectively. After drying in N_2 blow gas, the substrates were treated in a plasma cleaner for 15 min. A compact TiO_2 layer (c-TiO_2) was deposited onto FTO by spray pyrolysis of 1 mL titanium diisopropoxide bis(acetylacetonate) dissolved in 10 mL IPA at 500°C. After completing the spray pyrolysis step, the substrates were stored at 500 ℃ for 1 h to improve the electrical properties, slowly cooled to room temperature. Then, a mesoporous TiO_2 layer (m-TiO_2) was deposited by spin coating for 30 s at 3000 rpm, using a 30 nm particle paste (Dyesol 30 NR-D) diluted in ethanol/terpineol (78:22 w/w). The FTO/c-TiO_2 substrates prepared with m-TiO_2 were heated at 500 ℃ on a hot plate for 1 h and then slowly cooled to 200 ℃. The procedures of Li-doping of m-TiO_2 was following a published literature. To fabricate perovskite solar cells, 1.6 M perovskite precursor solution was prepared by mixing $FAPbI_3$ powder and MACl (35 mol%) in DMF and DMSO with a volume ratio of 4:1. Deposition of the perovskite onto FTO/c-TiO_2/m-TiO_2 substrate was achieved by spin-coating at 6,000 rpm for 30 s with 0.1 s ramping. During spin coating, 1 mL diethyl ether was dripped after spinning for 10 s using a pipette. The film was dried on a hot plate at 150 ℃ for 10 min. For ligand treatment, the 4HPA ligand powder was dissolved in IPA at 70 ℃ for 2 hours to form solutions with different concentrations (0.3, 0.6, 1, 2 and 3 mg mL^{-1}). Then 60 μl of the ligand solution was

dynamically spin-coated onto the perovskite films at 3000 rpm for 30 s and annealed at 80 ^oC for 5 minutes. The hole transport layer films (72.9 mg of Spiro-OMeTAD in 1 mL chlorobenzene with the addition of 17.5 μL of Li-TFSI solution (520 mg in 1 mL acetonitrile) and 29 μL of *t*-BP) were spin-coated at 3000 rpm for 30 s. Finally, a gold counter electrode (~80 nm) was deposited on the substrate using a thermal evaporation system.

Fabrication of solar modules: The modules consisted of eight solar cells connected in series using interconnects P1, P2, and P3 laser structuring. Three scribes, P1 (200 μm), P2 (200 μm), and P3 (400 μm) were accomplished by the laser beam with a power of 18, 10 and 7 W, respectively. To fabricate modules, a compact TiO_2 ETL was used. The large-area perovskite films were prepared by the blade coating of the perovskite precursors, and subjected to a vacuum-flash process and subsequent annealing. A 4~40-μL droplet of perovskite precursor was added into the gap (~200 μm) between the blade and substrate, and then the blade was moved on with a speed of ~200 mm s^{-1}. Next, the wet films were transferred into a vacuum chamber in a short time, and quickly pumped to ~10^2 Pa from atmospheric pressure within 15 s, and then maintained for 1 min at this pressure. After the vacuum-flash process, the substrate was transferred onto the hotplate for 20-min annealing at 150 ℃ and another 10-min heat-treatment at 100 °C. The perovskite deposition was carried out under ambient condition with relative humidity ~30%. Other procedures were consistent with the small-cell case. For the module encapsulation, modules were simply sealed with a cover glass and UV adhesive in a nitrogen-filled glove box, stored for 24 hours to achieve higher sealing effect, and the edges were sealed with 3M EVA tape or hot melt glue.

Characterizations: The UV-vis absorption spectra of deposited films were measured by a spectrophotometer (Agilent Cary 5000), the concentration of 4HPA in DMF solution is 0.03 mg mL^{-1}; In 4HPA: PbI_2 mixed solution, the concentration of 4HPA and PbI_2 are 0.03 and 0.001 mg mL^{-1}, respectively. Fourier transform infrared (FTIR) spectrum analysis was performed using an FTIR spectrometer (Thermo Scientific Nicolet iS50), the 4HPA: PbI_2 mixed powders were prepared by the following procedures: 1) 4HPA and PbI_2 at a mass ratio of 2:1 were added into DMF and stirring for 1 h at room temperature; 2) the DMF in the mixed solution was then removed in a rotary evaporator to obtain the solid powder required for testing. The X-ray photoelectron spectrum (XPS) was performed using an X-ray photoelectron spectroscopy system (Axis Supra, Shimadzu) with Al Kα X-ray radiation (1486.6 eV) as the X-ray source. Current-voltage characteristics were recorded on a solar simulator under AM 1.5 G standard light (100 mW cm^{-2}) equipped with a Keithley 2400 source meter. IPCE spectra were measured on an IPCE system (Newport) equipped with a

xenon lamp (Newport, 300 W xenon lamp), a monochromator (Cornerstone 260, Newport) and a power meter (2936-R, Newport). The steady-state and time-resolved photoluminescence spectra were performed at Edinburgh Instruments (FLS980) with an excitation wavelength of 450 nm. Water contact angles were measured on an optical contact-angle meter system. Scanning electron microscope (SEM, Hitachi S-4800) were applied to record the film morphology. X-ray photoelectron spectroscopy (XPS) was performed on a Thermo Scientific ESCA Lab 250Xi. Ultraviolet photoelectron spectroscopy (UPS) measurement was performed on a PHI5000 Versa Probe III (Scanning ESCA Microprobe) instrument. The electrochemical impedance spectroscopy (EIS) was performed using a Chi760e electrochemical workstation.

DFT calculations: The first principle calculations are performed to reveal the mechanism of NO2RR by using the Vienna ab initio simulation package. The program has the projected enhancement wave pseudopotential and the generalized gradient approximation of Perdew, Burke and Enzzerhof (PBE) exchange correlation functional, which is used to optimize the structure and obtain the free energy of all structures. The cutoff energy of the plane wave basis set is 500 eV and a Monkhorst-Pack mesh of 3×3×1 is used in K-sampling in the adsorption energy calculation. In addition, van der Waals interactions corrected using DFT-D2 considered. The electronic self-consistent iteration is set to 10^{-5} eV, and the positions of all of the atoms are fully relaxed until the residual force on each atom is below 0.02 eV $Å^{-1}$. 15 Å of vacuum layer along the z-direction is applied to avoid periodic interactions. The free energy of structure A (GA) is calculated by GA=EA+ZPE-TS: the total energy (EA), zero-point energy (ZPE), T is the temperature (298.15 K), and the entropy (S) of structure A.

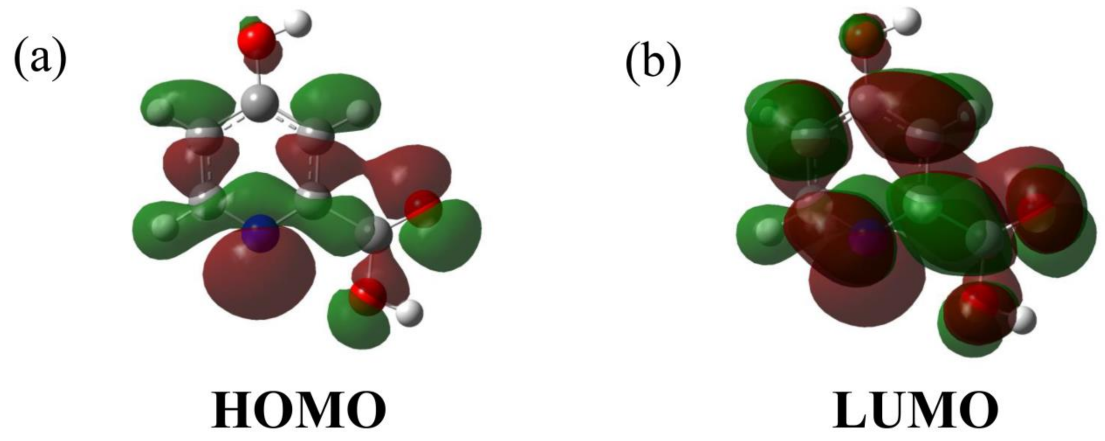

Figure S1. Optimized molecular configurations and corresponding (a) HOMO and (b) LUMO profiles of 4HPA.

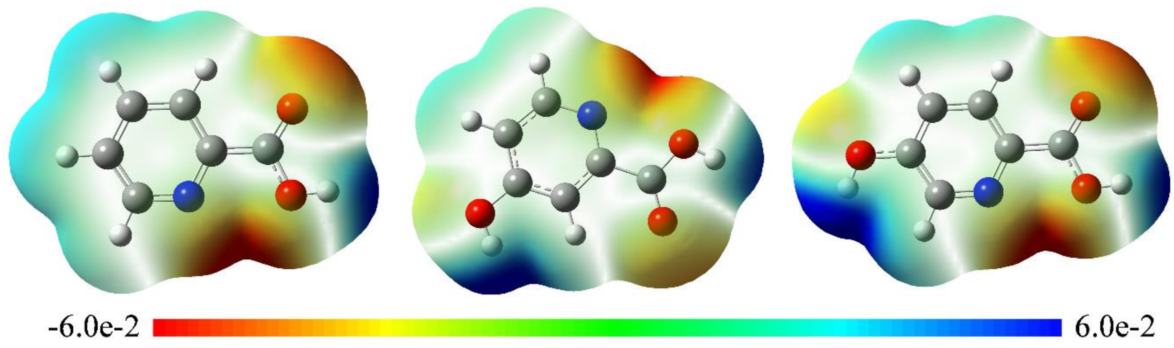

Figure S2. Electrostatic potential (ESP) plots of 2-picolinic acid, 4-hydroxypicolinic acid (4HPA) and 5-hydroxypicolinic acid (5HPA).

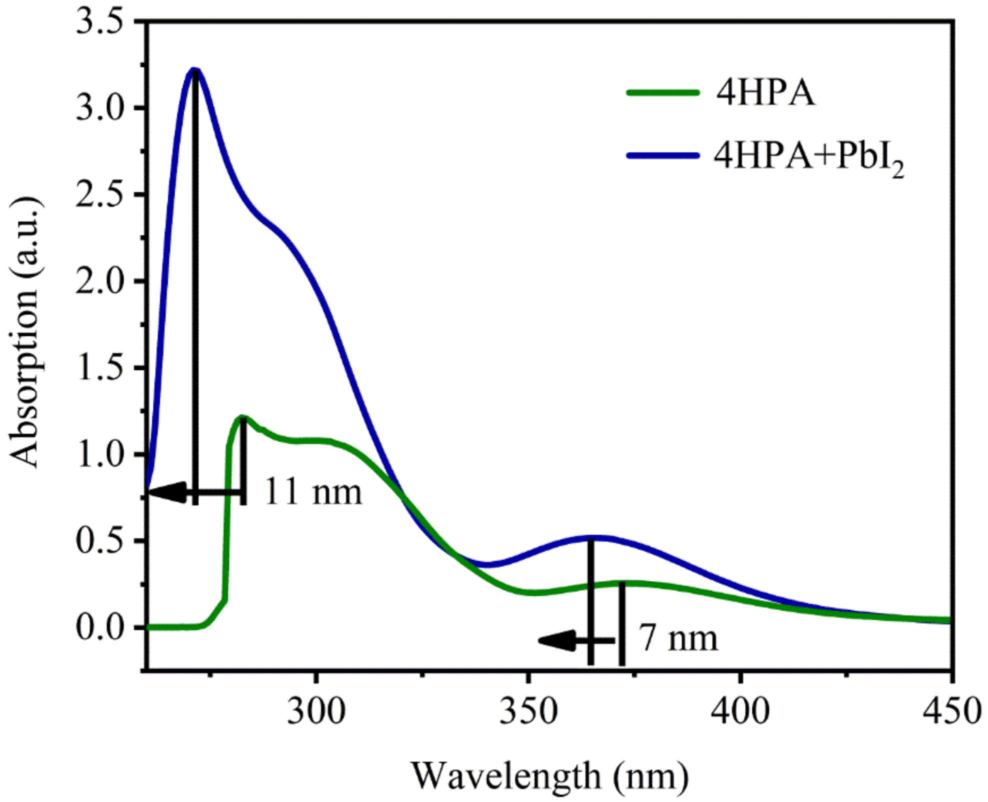

Figure S3. UV-vis absorption spectra of 4HPA and 4HPA:PbI_2 solutions.

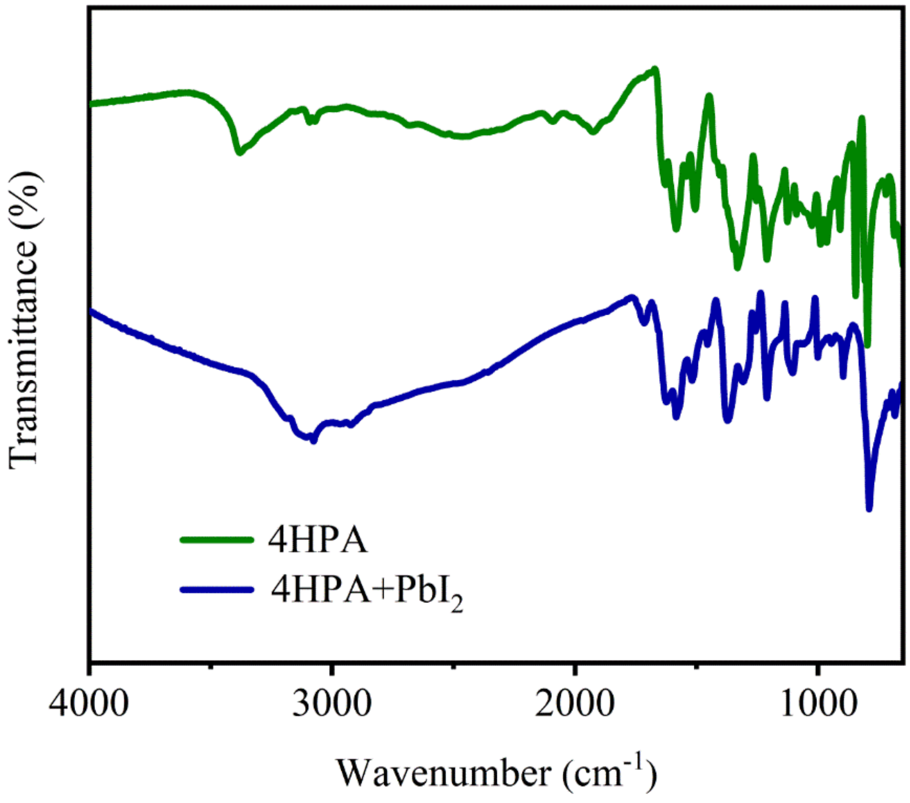

Figure S4. FTIR of the powder of 4HPA and 4HPA: PbI_2 blend.

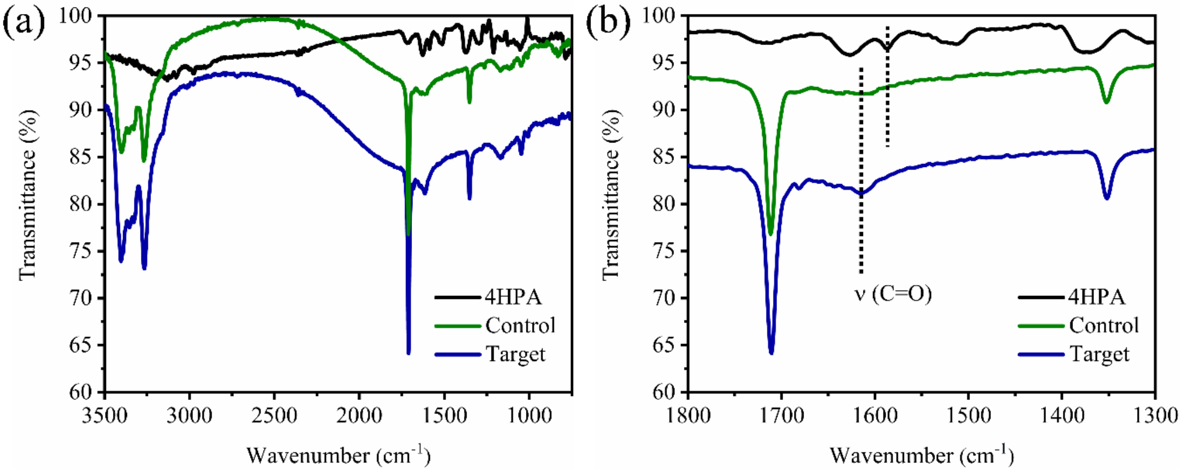

Figure S5. (a) FTIR spectra of the ligand, control, and target films; (b) the local magnification curve of FTIR spectra.

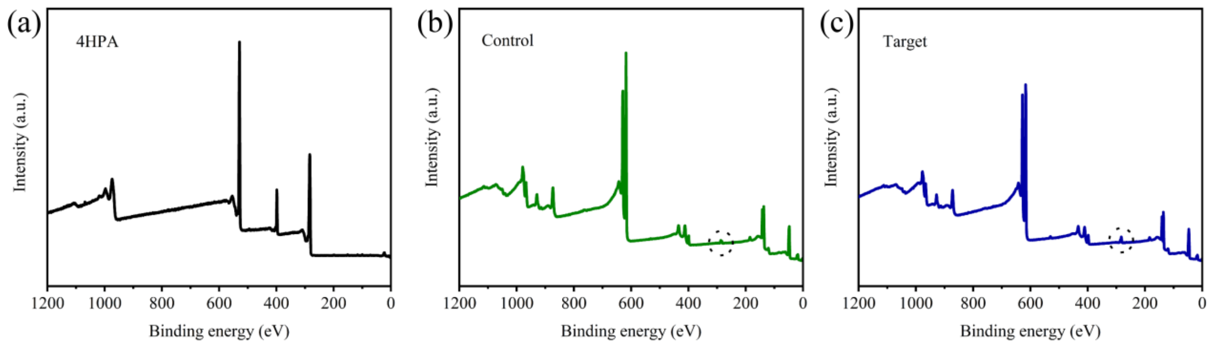

Figure S6. The entire XPS spectra of (a) 4HPA, (b) control film, and (c) target film.

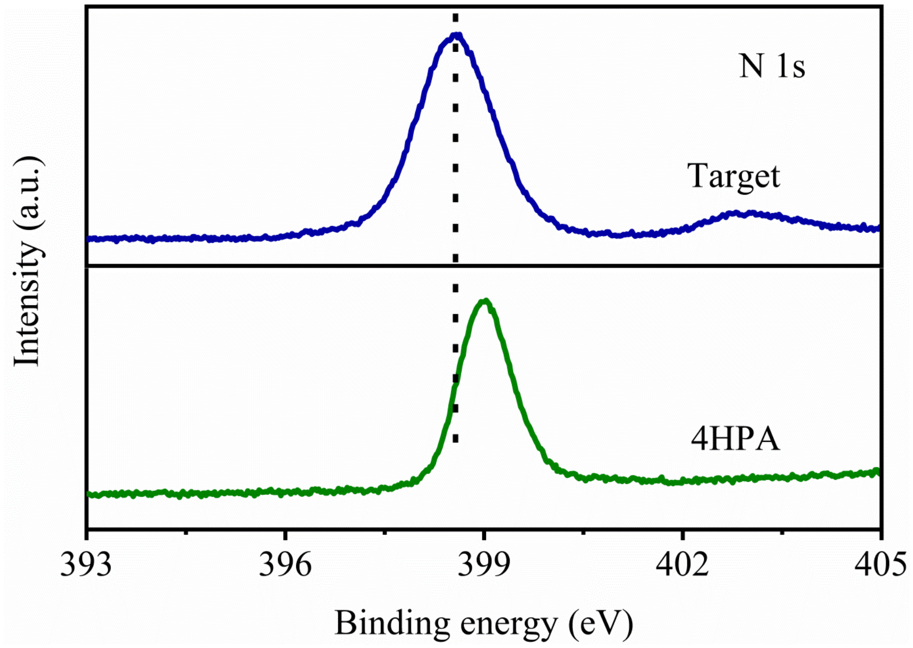

Figure S7. XPS spectra of N 1s for pure 4HPA and 4HPA treated perovskite film.

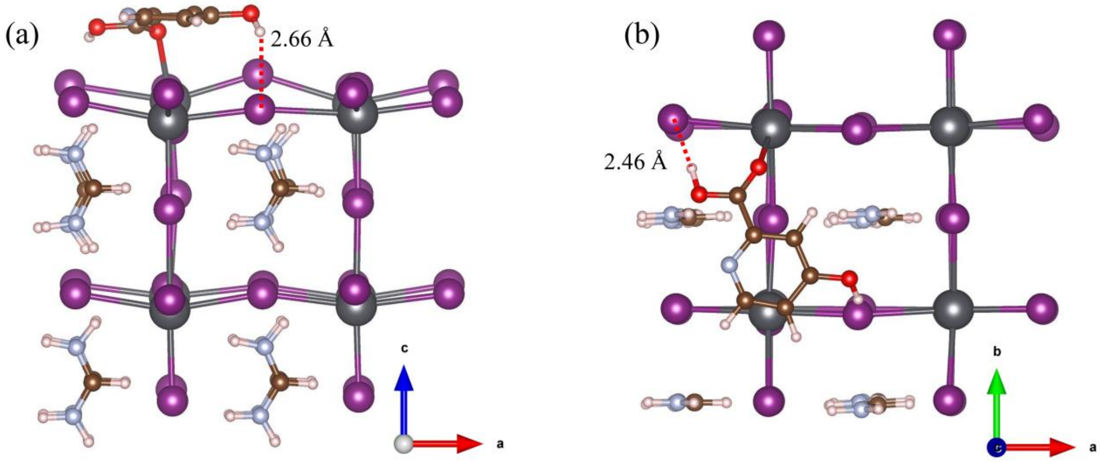

Figure S8. (a) The side view and (b) top view of the 4HPA ligand in the optimized molecular geometries arranged on the surface of perovskite film.

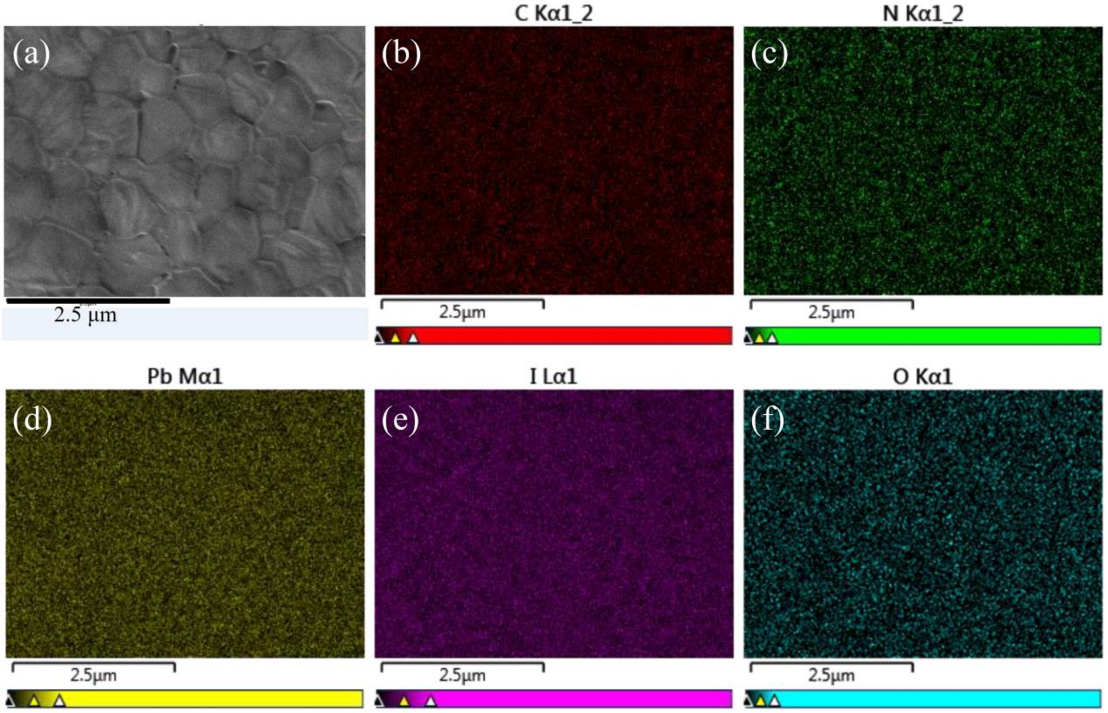

Figure S9. (a) Top-view SEM image of the target perovskite film. SEM-EDS mappings of C (b), N (c), Pb (d), I (e), and O (f) for target film deposited onto the FTO substrate.

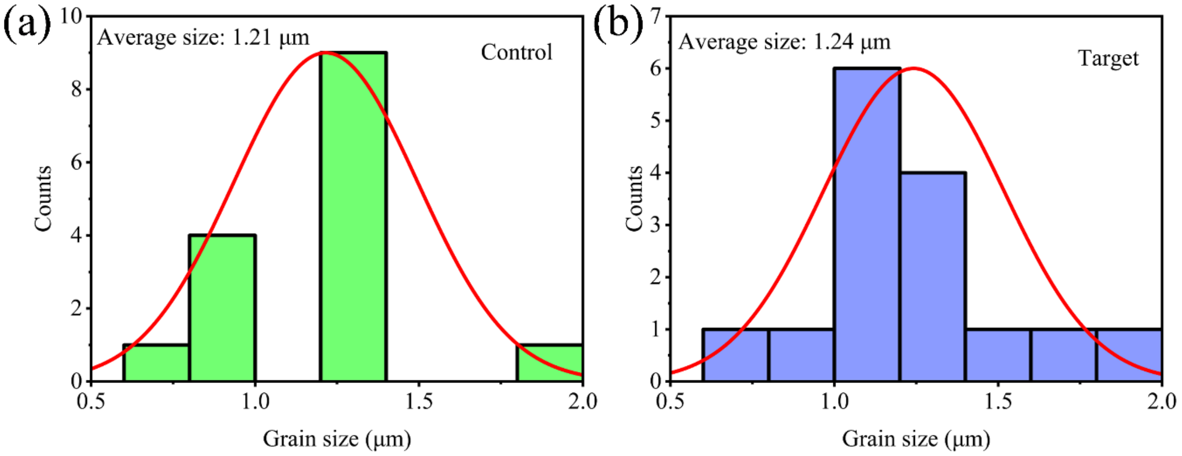

Figure S10. Grain size distribution of perovskite films without or with 4HPA treatment.

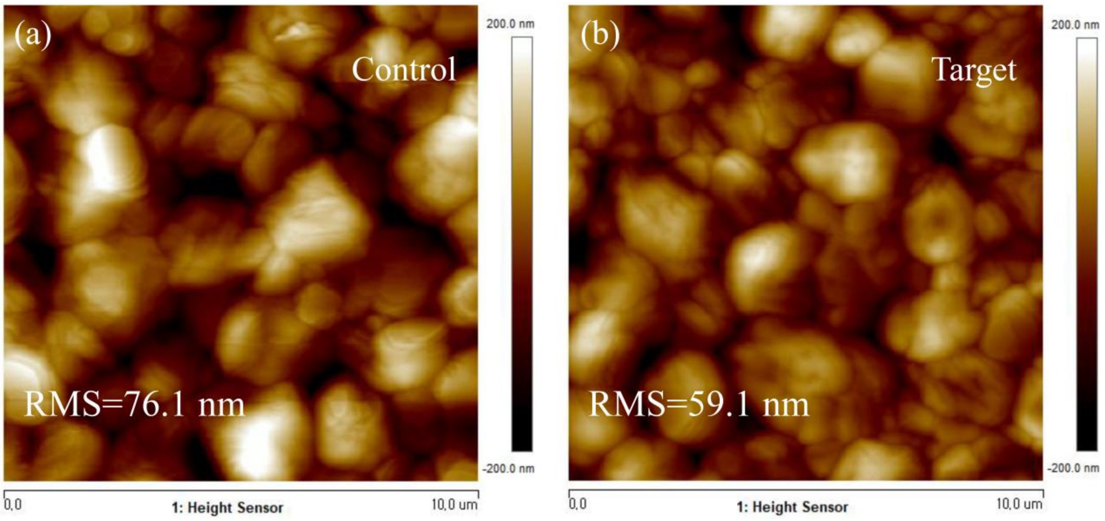

Figure S11. Surface AFM topographies of control (a) and target (b) perovskite films.

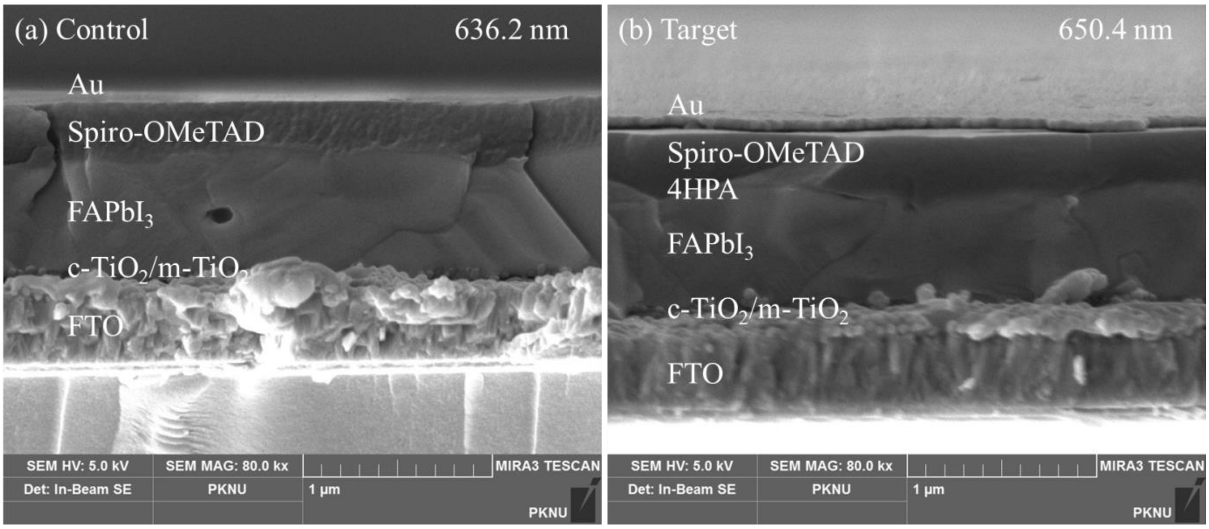

Figure S12. Cross-sectional SEM images of PeSCs with control and 4HPA-treated films.

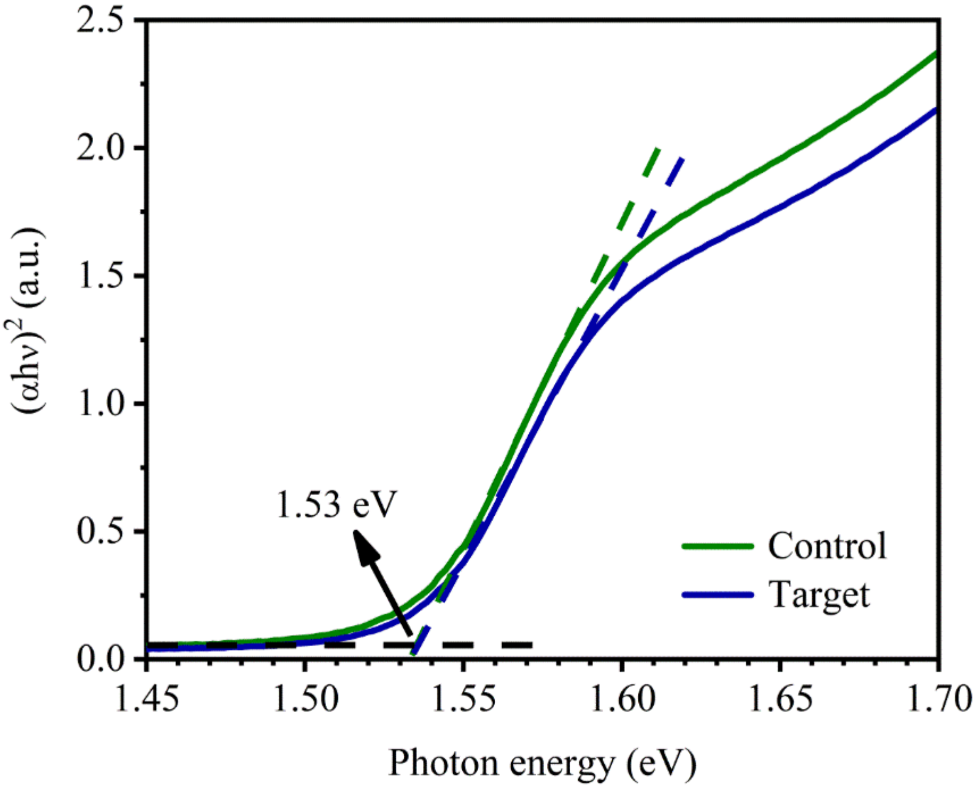

Figure S13. Tauc plot of the perovskite films on glass substrate.

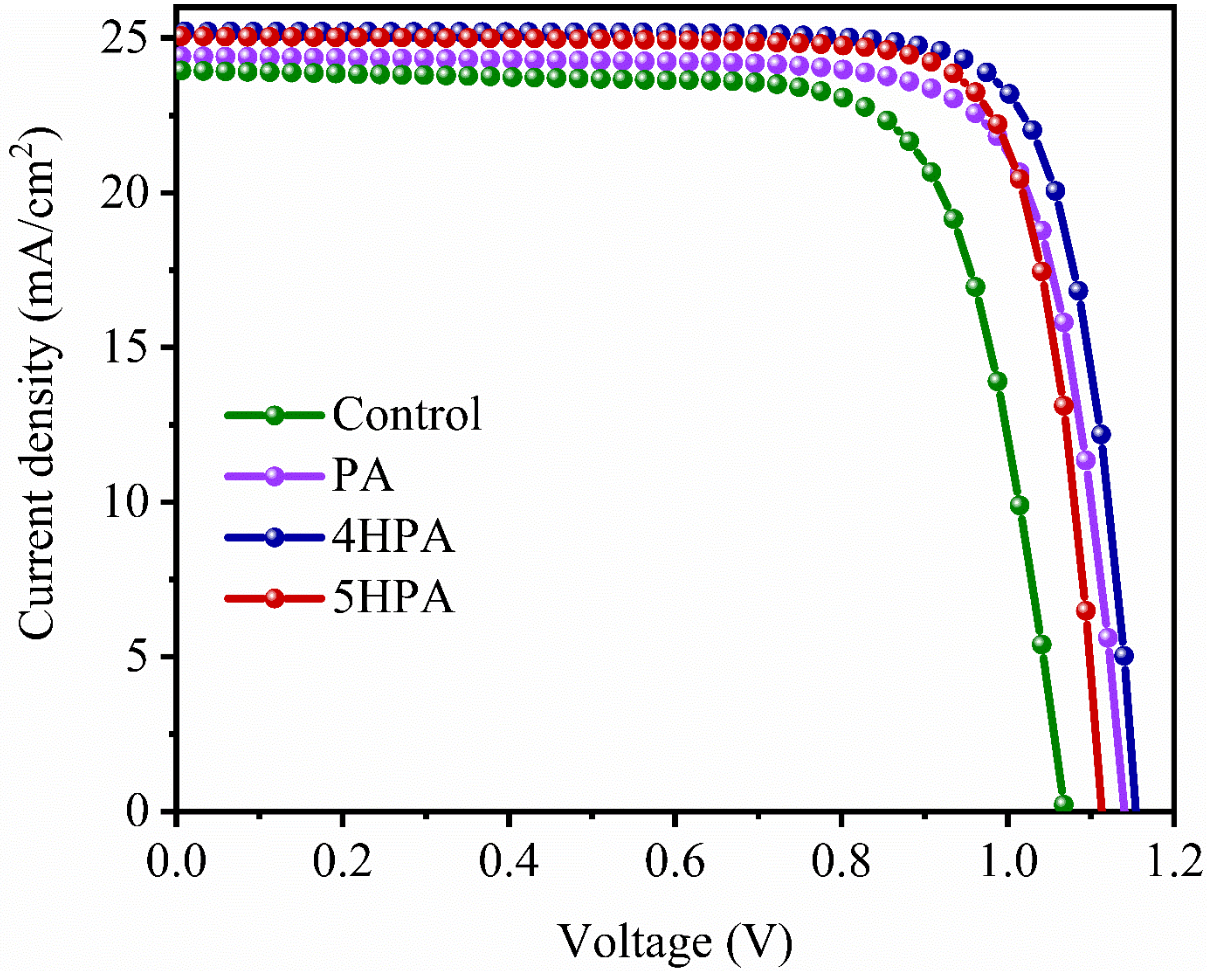

Figure S14. Current *J*-*V* curves of the four PeSCs under AM 1.5G irradiation (100 mW/cm^2).

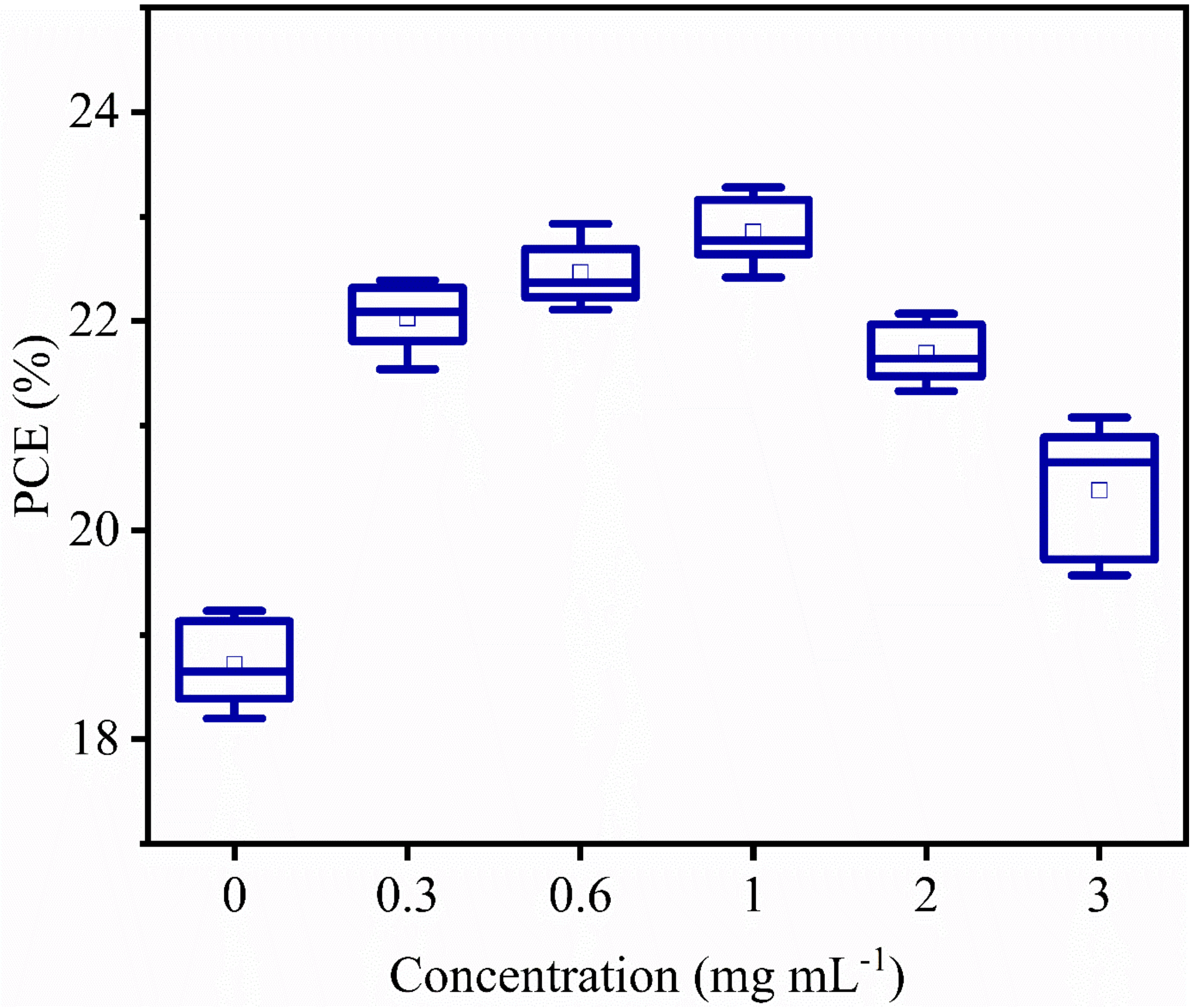

Figure S15. The PCE distribution of the PeSCs with different concentrations of 4HPA treatment.

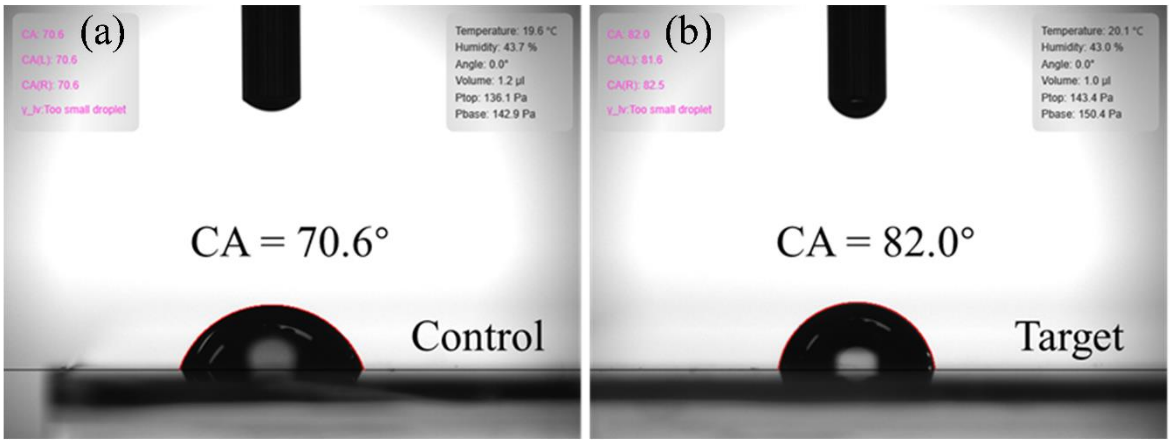

Figure S16. Static contact angle measurements with water on top of different perovskite films.

Table S1. Photovoltaic parameters of the best and average for the devices of control and after post-treatment with different ligands. [a)]

Sample	Area [cm^2]	*J*sc [mA/cm^2]	*V*oc [V]	FF [%]	PCE [%]
Control	0.12	24.05 (23.82)	1.06 (1.05)	75.21 (74.84)	19.23 (18.72)
PA	0.12	24.42 (24.19)	1.14 (1.12)	77.81 (76.12)	21.64 (20.64)
4HPA	0.12	25.21 (25.08)	1.15 (1.15)	80.11 (79.18)	23.28 (22.85)
5HPA	0.12	25.06 (24.95)	1.11 (1.10)	80.14 (79.25)	22.33 (21.81)

[a)] The average values are calculated from 20 devices.

Table S2. TRPL fitting parameters of the perovskite films. The data was fitted using bi-exponential decay equation $y = A_1\exp(-x/\tau_1) + A_2\exp(-x/\tau_2) + y_0$, in which τ_1 is time constant for fast decay component and τ_2 is time constant for slow decay component. Average lifetime was estimated from the equation $\tau_{ave} = (A_1{\tau_1}^2 + A_2{\tau_2}^2)/(A_2\tau_2 + A_2\tau_2)$. The TRPL was excited using 450 nm wavelength laser.

Perovskite film	A_1	T_1 (ns)	A_2	T_2 (ns)	T_{ave} (ns)
Control	191.86	8.04	824.15	317.91	158.27
Target	68.20	19.09	874.89	552.86	539.03

Table S3. Statistical photovoltaic parameters of the PeSCs with various concentrations of 4HPA treatment.

Concentration [mg/mL]		Jsc [mA/cm^2]	Voc [V]	FF [%]	PCE [%]
Control	Champion	24.05	1.06	75.21	19.23
	Average [a)]	23.82 ± 0.43	1.05 ± 0.04	74.84 ± 1.83	18.72 ± 0.77
0.3	Champion	25.07	1.13	78.44	22.39
	Average	24.79 ± 0.49	1.13 ± 0.03	77.64 ± 1.74	21.75 ± 0.74
0.6	Champion	25.14	1.14	79.39	22.93
	Average	24.86 ± 0.55	1.13 ± 0.04	78.60 ± 2.12	22.08 ± 0.62
1.0	Champion	25.21	1.15	80.11	23.28
	Average	25.08 ± 0.22	1.15 ± 0.02	79.18 ± 1.42	22.85 ± 0.43
2.0	Champion	24.54	1.13	79.23	22.07
	Average	24.51 ± 0.35	1.11 ± 0.03	78.76 ± 1.64	21.43 ± 0.68
3.0	Champion	24.46	1.12	76.90	21.08
	Average	23.98 ± 0.46	1.11 ± 0.03	76.57 ± 1.79	20.38 0.75

[a)] The average values are calculated from 20 devices.

Table S4. Reported performance characteristics of $FAPbI_3$ PeSCs stabilized with organic molecules.

Year	PCE [%]	*J*sc [mA/cm^2]	*V*oc [V]	FF [%]	Strategy	Ref.
2019	18.94	25.1	1.08	70.0	AVAI additive	Ref.[2]
2020	21.20	23.80	1.18	75.2	SM2 post-treated	Ref.[3]
2021	20.90	24.34	1.13	75.7	Poly(D) antisolvent	Ref.[4]
2021	22.39	24.80	1.15	78.5	TMTA antisolvent	Ref.[5]
2022	20.90	25.54	1.12	73.09	PAH post-treated	Ref.[6]
2022	22.59	24.24	1.15	80.0	PVP-I Additive	Ref.[7]
2022	23.80	24.90	1.15	83.0	1,8-ND post-treated	Ref.[8]
2023	23.28	25.21	1.15	80.1	4HPA post-treated	This work

References

[1] M. Kim, J. Jeong, H. Lu, T. K. Lee, F. T. Eickemeyer, Y. Liu, I. W. Choi, S. J. Choi, Y. Jo, H.-B. Kim, S.-I. Mo, Y.-K. Kim, H. Lee, N. G. An, S. Cho, W. R. Tress, S. M. Zakeeruddin, A. Hagfeldt, J. Y. Kim, M. Grätzel, D. S. Kim, *Science* **2022**, *375*, 302.

[2] A. Q. Alanazi, D. J. Kubicki, D. Prochowicz, E. A. Alharbi, M. E. F. Bouduban, F. Jahanbakhshi, M. Mladenovic, J. V. Milic, F. Giordano, D. Ren, A. Y. Alyamani, H. Albrithen, A. Albadri, M. H. Alotaibi, J. E. Moser, S. M. Zakeeruddin, U. Rothlisberger, L. Emsley, M. Gratzel, *J. Am. Chem. Soc.* **2019**, *141*, 17659.

[3] K. Wang, J. Liu, J. Yin, E. Aydin, G. T. Harrison, W. Liu, S. Chen, O. F. Mohammed, S. De Wolf, *Adv. Funct. Mater.* **2020**, *30,* 2002861.

[4] Z. Liu, F. Liu, C. Duan, L. Yuan, H. Zhu, J. Li, Q. Wen, G. I. N. Waterhouse, X. Yang, K. Yan, *Chem. Eng. J.* **2021**, *419,* 129482.

[5] H. Zhang, Z. Chen, M. Qin, Z. Ren, K. Liu, J. Huang, D. Shen, Z. Wu, Y. Zhang, J. Hao, C. S. Lee, X. Lu, Z. Zheng, W. Yu, G. Li, *Adv. Mater.* **2021**, *33*, e2008487.

[6] J. Tang, L. Liu, Z. Yu, J. Du, X. Cai, M. Zhang, M. Zhao, L. Bai, Z. Gai, S. Cui, X. Li, T. Jiu, *Adv. Sustain. Syst.* **2022**, *6,* 2100510.

[7] D. H. Kang, C. Ma, N. G. Park, *ACS Appl. Mater. Inter.* **2022**, *14*, 8984.

[8] G. Li, J. Song, J. Wu, Y. Xu, C. Deng, Z. Song, X. Wang, Y. Du, Q. Chen, R. Li, W. Sun, Z. Lan, *Chem. Eng. J.* **2022**, *449,* 137806.
